# Serum Malondialdehyde-Modified Low-Density Lipoprotein as a Risk Marker for Peripheral Arterial Stiffness in Maintenance Hemodialysis Patients

**DOI:** 10.3390/medicina60050697

**Published:** 2024-04-24

**Authors:** Wei-Nung Liu, Yi-Chiung Hsu, Chia-Wen Lu, Ssu-Chin Lin, Tsung-Jui Wu, Gen-Min Lin

**Affiliations:** 1 Department of Medicine, Hualien Armed Forces General Hospital, Hualien 97144, Taiwan; weinungliu@gmail.com (W.-N.L.); noirwen@gmail.com (C.-W.L.); 2 Department of Biomedical Sciences & Engineering, National Central University, Taoyuan 320317, Taiwan; syic@ncu.edu.tw; 3 Department of Medicine, Tri-Service General Hospital, National Defense Medical Center, Taipei 11490, Taiwan; 4 Institute of Medical Sciences, Tzu Chi University, Hualien 97004, Taiwan; siching.lin@gmail.com; 5 Department of Nursing, Hualien Armed Forces General Hospital, Hualien 97144, Taiwan

**Keywords:** cardiovascular disease, end-stage renal disease, hemodialysis, malondialdehyde-modified low-density lipoprotein, peripheral arterial stiffness, pulse wave velocity

## Abstract

*Background and Objectives*: Peripheral arterial stiffness (PAS), assessed by brachial-ankle pulse wave velocity (baPWV), is an independent biomarker of cardiovascular diseases (CVD) in patients on maintenance hemodialysis (HD). Malondialdehyde-modified low-density lipoprotein (MDA-LDL), an oxidative stress marker, has been linked to atherosclerosis and CVD. However, the association between serum MDA-LDL and PAS among HD patients has not been fully elucidated. This study aimed to examine the association of serum MDA-LDL with PAS in HD patients and to identify the optimal cutoff value of serum MDA-LDL for predicting PAS. *Materials and Methods*: A cross-sectional study was conducted in 100 HD patients. Serum MDA-LDL was quantified using an enzyme-linked immunosorbent assay (ELISA), and baPWV was measured using a volume plethysmographic device. Patients were divided into the PAS group (baPWV > 18.0 m/s) and the non-PAS group (baPWV ≤ 18.0 m/s). The associations of baPWV and other clinical and biochemical parameters with serum MDA-LDL were assessed by multivariable logistic regression analyses. A receiver operating characteristic (ROC) curve analysis was performed to determine the optimal cutoff value of serum MDA-LDL for predicting PAS. *Results*: In multivariable logistic regression analysis, higher serum MDA-LDL, older age, and higher serum C-reactive protein [odds ratios (ORs) and 95% confidence intervals: 1.014 (1.004–1.025), 1.044 (1.004–1.085) and 3.697 (1.149–11.893)] were significantly associated with PAS. In the ROC curve analysis, the optimal cutoff value of MDA-LDL for predicting PAS was 80.91 mg/dL, with a sensitivity of 79.25% and a specificity of 59.57%. *Conclusions*: Greater serum MDA-LDL levels, particularly ≥80.91 mg/dL, were independently associated with PAS in HD patients. The findings suggest that oxidative stress plays a crucial role in the pathogenesis of PAS, and targeting MDA-LDL may be a potential therapeutic strategy for reducing cardiovascular risk in HD patients.

## 1. Introduction

Cardiovascular disease (CVD) is the primary cause of morbidity and mortality among patients with end-stage renal disease (ESRD) receiving maintenance hemodialysis (HD) [1,2,3]. The increased cardiovascular risk in this population is attributed to a complex interplay of traditional risk factors, e.g., hypertension, diabetes mellitus, and dyslipidemia, and non-traditional risk factors, including oxidative stress, inflammation, and mineral bone disorders [4,5,6]. Identifying novel biomarkers and therapeutic targets for CVD in HD patients is crucial for improving their clinical outcomes and quality of life.

Peripheral arterial stiffness (PAS), characterized by reduced arterial elasticity with increased pulse wave velocity (PWV), has emerged as an independent predictor of cardiovascular events and all-cause mortality in HD patients [7,8]. Brachial-ankle PWV (baPWV), a non-invasive measure of arterial stiffness, has been widely used to assess PAS in HD patients [9]. Several factors such as old age, hypertension, diabetes mellitus, and chronic inflammation, have been associated with higher baPWV among HD patients [10,11,12]. However, the role of oxidative stress in the pathogenesis of PAS in this population remains poorly understood.

In the general population, triglycerides (TG), among the blood lipid parameters, have been revealed to have the strongest associations with PAS, while low-density lipoprotein cholesterol (LDL-C) levels have been more strongly associated with atherosclerosis than with PAS [13]. Notably, a few modified LDLs, such as oxidized LDL (oxLDL), were found to be associated with both atherosclerosis and PAS [14]. Malondialdehyde (MDA)-modified LDL, a biomarker of oxidative stress, is another kind of modified LDL that is formed by the reaction of MDA with the amino groups of apolipoprotein B in LDL particles [15,16]. In prior population-based studies, MDA-LDL has been implicated in the development of atherosclerosis and clinical CVD in the general population [16,17,18]. In experimental studies, MDA-LDL was observed to promote endothelial dysfunction, vascular inflammation, and foam cell formation, subsequently leading to the progression of atherosclerotic lesions [16,19,20]. Additionally, elevated serum MDA-LDL has been associated with a higher risk of PAS in patients with hypertension or diabetes mellitus [13,21,22]. However, in HD patients, there were rare studies to examine the effects of various lipids on aortic stiffness and PAS. To the best of our knowledge, MDA-LDL was the most commonly studied lipid target in HD patients, which has been found to be associated with aortic wall stiffness [21], whereas its relationship with PAS among HD patients has not been fully elucidated. Since both aortic wall stiffness and PAS have been recognized as crucial risk factors of clinical CVD in HD patients, the present study aimed to examine the association between serum MDA-LDL levels and PAS, as assessed by baPWV, in a sample of HD patients. We hypothesized that elevated serum MDA-LDL levels would be independently associated with increased baPWV, and that serum MDA-LDL levels could serve as a useful biomarker for predicting PAS in this high-risk population.

To investigate our hypotheses, we performed a cross-sectional study involving 100 HD patients. We assessed the associations between serum MDA-LDL levels, baPWV, and other pertinent clinical and biochemical parameters. Additionally, we aimed to determine the optimal cutoff value of serum MDA-LDL levels for predicting PAS in this population. Our results may offer novel insights into the role of oxidative stress in PAS pathogenesis and emphasize the potential utility of serum MDA-LDL as an innovative marker and therapeutic target for CVD in HD patients.

## 2. Materials and Methods

### 2.1. Participants and Study Protocol

**Figure 1:**
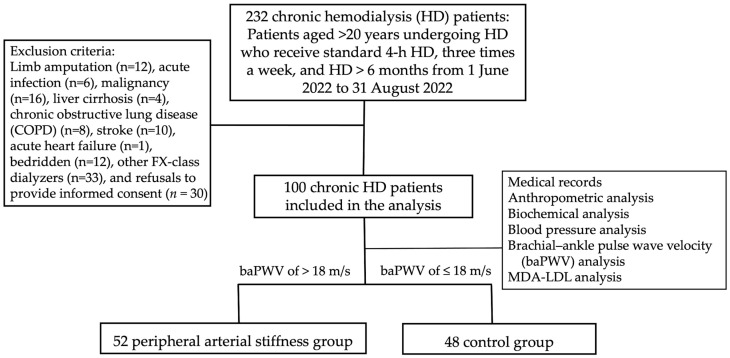
Flowchart of the selection of HD patients receiving measurements of both brachial-ankle pulse wave velocity and Malondialdehyde (MDA)-modified LDL.

This observational study was conducted at a single dialysis center in eastern Taiwan from June to August 2022. According to the patient enrollment flowchart (Figure 1), there were 232 adult hemodialysis patients who had received maintenance hemodialysis for more than 3 months at the time of screening. Patients who had a limb amputated (*n* = 12), acute infection (*n* = 6), malignancy (*n* = 16), liver cirrhosis (*n* = 4), chronic obstructive lung disease (COPD) (*n* = 8), stroke (*n* = 10), acute heart failure (*n* = 1) or were bedridden (*n* = 12) were excluded. Patients who used other than a high-flux dialyzer (*n* = 33) or refused to provide informed consent (*n* = 30) were also excluded.

The study enrolled 100 patients who met the following criteria: age ≥ 20 years, receiving regular HD sessions (4 h per session, 3 times per week) for more than 3 months, and using high-flux dialyzers (FX-class, Fresenius Medical Care, Germany). Patients’ demographic and clinical information, including the presence of comorbidities, e.g., diabetes mellitus and hypertension, was collected from their medical records. The study protocol was reviewed and approved by the Tzu Chi Hospital Institutional Review Board (IRB108–219-A) and adhered to the principles of the Declaration of Helsinki. Informed written consent was obtained from all study participants.

### 2.2. Assessment of Anthropometric Parameters

Anthropometric measurements were carried out by a trained staff member, with patients dressed in lightweight clothing and barefoot. Body weight and height were measured and rounded up to the nearest 0.5 kg and 0.5 cm, respectively. Body mass index (BMI) was calculated by dividing the participant’s weight in kilograms by the square of the participant’s height in meters.

### 2.3. Laboratory Investigations

Following an overnight fast of 8–12 h, blood samples (approximately 5 mL) were drawn from each participant and centrifuged at 3000× *g* for 10 min. An automated analyzer (Siemens Advia 1800; Siemens Healthcare GmbH, Henkestr, Germany) was employed to measure serum concentrations of blood urea nitrogen (BUN), creatinine, total cholesterol, triglycerides (TG), glucose, total calcium, phosphorus, and C-reactive protein (CRP). Enzyme-linked immunosorbent assay kits were used to determine serum levels of intact parathyroid hormone (iPTH) (Abcam, Cambridge, MA, USA) and MDA-LDL (Sekisui Diagnostics GmbH, Kaplaneigasse, Pfungstadt, Germany) [21].

### 2.4. Evaluation of Blood Pressure and Brachial-Ankle Pulse Wave Velocity

Following blood sample collection, patients rested in a supine position for 10 min. The trained staff member took the participants’ morning blood pressure (BP) of the upper arm using an automatic oscillometric device. Three measurements of systolic BP (SBP) and diastolic BP (DBP) were taken at 5-min intervals, and the mean values were used for analysis. Hypertension was defined as SBP ≥ 140 mmHg, DBP ≥ 90 mmHg, or the use of antihypertensive medications for at least two weeks, based on the Eighth Joint National Committee (JNC 8) guidelines. Assessment of baPWV was performed by a volume plethysmographic device (VP-2000, Omron, Japan) with four pneumatic cuffs coupled with oscillometric and plethysmographic sensors wrapped around the upper arms and ankles [23]. Patients with left or right baPWV values > 18.0 m/s were classified as the PAS group, according to the cutoff value established by the Physiological Diagnosis Criteria for Vascular Failure Committee of Japan [24,25].

### 2.5. Data Analysis

The normality of data distribution was assessed using the Kolmogorov-Smirnov test. Normally distributed continuous variables were expressed as mean ± standard deviation and compared using the two-tailed Student’s independent *t*-test. Non-normally distributed variables, including TG, glucose, iPTH, and CRP levels, were presented as median and interquartile range and compared using the Mann-Whitney U test. These non-normally distributed variables were log-transformed to achieve normality for further analysis. The chi-square test was used to analyze categorical variables. The associations between serum MDA-LDL and other variables were assessed using Spearman’s rank-order correlation coefficient. Variables significantly correlated with PAS were then included in a multivariable logistic regression analysis. A receiver operating characteristic (ROC) curve was constructed to identify the optimal cutoff value of serum MDA-LDL for predicting PAS, and the area under the curve (AUC) was calculated to assess its predictive ability. Statistical analyses were performed using IBM SPSS Version 19.0 (SPSS, Inc., Chicago, IL, USA), with statistical significance set at *p* < 0.05.

## 3. Results

Demographic, clinical, and biochemical characteristics of the study population are shown in Table 1. The study comprised 100 HD patients with a mean age of 63.84 ± 13.51 years. The median HD duration was 55.92 months (interquartile range [IQR]: 21.96–123.60 months). Of the patients, 47 (47.0%) were women, 46 (46.0%) had diabetes mellitus, and 46 (46.0%) had hypertension. The mean left and right baPWV were 18.04 ± 3.25 m/s and 18.14 ± 3.29 m/s, respectively. The median serum MDA-LDL level was 88.67 mg/dL (IQR: 69.96–148.52 mg/dL).

Compared to the non-PAS group (*n* = 48), the PAS group (*n* = 52) were older (67.37 ± 12.11 vs. 57.94 ± 13.35 years, *p* < 0.001), had lower serum albumin levels (4.06 ± 0.43 vs. 4.25 ± 0.41 g/dL, *p* = 0.022), lower serum creatinine levels (8.99 ± 1.67 vs. 9.85 ± 1.98 mg/dL, *p* = 0.021), higher serum MDA-LDL levels (119.67 [81.65–176.54] vs. 78.38 [59.41–98.35] mg/dL, *p* < 0.001), and higher serum CRP levels (0.35 [0.10–1.01] vs. 0.16 [0.05–0.40] mg/dL, *p* = 0.007).

Table 2 demonstrated the multivariable logistic regression analysis results that higher serum MDA-LDL levels (odds ratio [OR] =1.014, 95% confidence interval [CI]: 1.004–1.025, *p* = 0.009), older age (OR = 1.044, 95% CI: 1.004–1.085, *p* = 0.031), and higher serum CRP levels (OR = 3.697, 95% CI: 1.149–11.893, *p* = 0.028) were independently associated with PAS in HD patients.

Table 3 shows the correlation between baPWV, serum MDA-LDL, and clinical variables. Spearman’s rank-order correlation analysis revealed that left baPWV values were positively correlated with age (r = 0.284, *p* = 0.004), serum MDA-LDL levels (r = 0.385, *p* < 0.001), log-transformed glucose levels (r = 0.227, *p* = 0.023), and log-transformed CRP levels (r = 0.307, *p* = 0.002) and negatively correlated with serum creatinine levels (r = −0.221, *p* = 0.027). Right baPWV values were positively correlated with age (r = 0.308, *p* = 0.002), serum MDA-LDL levels (r = 0.390, *p* < 0.001), and log-transformed CRP levels (r = 0.249, *p* = 0.013) and negatively correlated with serum creatinine levels (r = −0.216, *p* = 0.031). Log-transformed serum MDA-LDL levels were positively correlated with waist circumference (r = 0.225, *p* = 0.025), total cholesterol (r = 0.218, *p* = 0.029), and log-transformed CRP levels (r = 0.197, *p* = 0.049).

**Figure 2:**
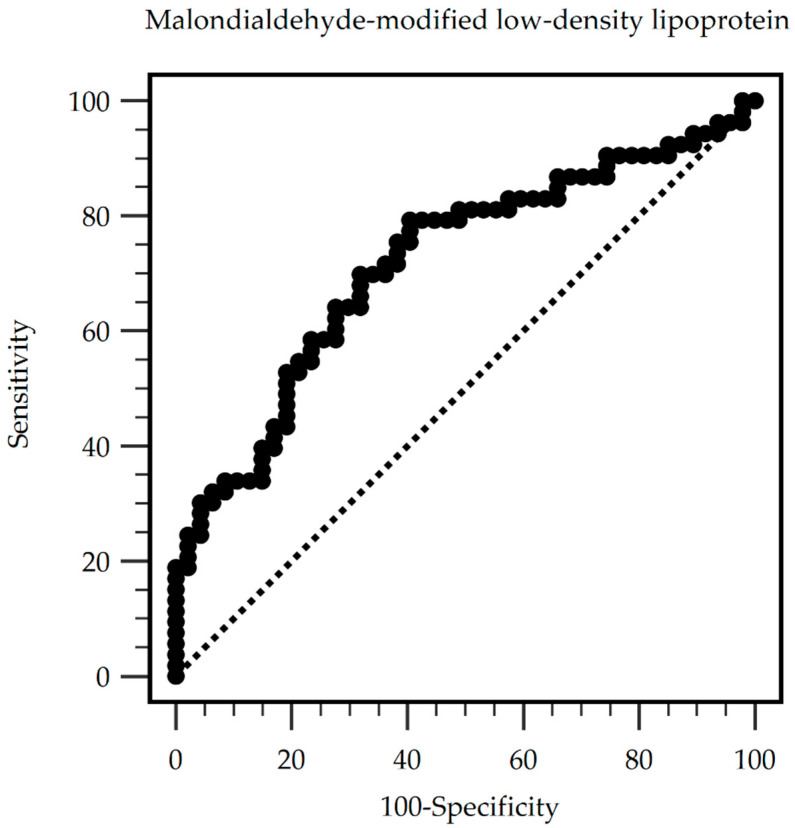
Peripheral arterial stiffness can be predicted by serum malondialdehyde-modified low-density lipoprotein levels among chronic hemodialysis patients, as indicated by the area under the receiver operating characteristic curve. Area under the ROC curve (AUC): 0.717. 95% Confidence interval: 0.618–0.803. *p* < 0.001. Cut-off level 80.91 mg/dL. (Sensitivity: 79.25%; specificity: 59.57%; positive predictive values: 68.85%; and negative predictive values: 71.80%).

The predictive value of serum MDA-LDL levels for PAS. The ROC curve analysis reveals that the optimal cut-off value of serum MDA-LDL for predicting PAS among HD patients was 80.91 mg/dL, with a sensitivity of 79.25%, specificity of 59.57%, positive predictive value of 68.85%, and negative predictive value of 71.80% (Table 4). The area under the ROC curve was 0.717 (95% CI: 0.618–0.803, *p* < 0.001) (Figure 2).

## 4. Discussion

In this cross-sectional study, we investigated the association between serum MDA-LDL levels and PAS among HD patients. Using multivariable logistic regression analysis, we found that higher serum MDA-LDL levels, older age, and elevated serum CRP levels were independently associated with PAS in this population. Furthermore, ROC curve analysis revealed that serum MDA-LDL levels greater than 80.91 mg/dL could potentially predict PAS in HD patients.

Our findings highlight the association between serum MDA-LDL levels and PAS in HD patients, suggesting that MDA-LDL potentially plays a role in the development of PAS in this population. Oxidative stress as reflected by serum MDA-LDL levels plays an important role in the pathogenesis of arterial stiffness and atherosclerosis [26,27]. MDA-LDL, a surrogate of oxidative stress, has been associated with endothelial dysfunction and atherosclerosis in various populations [28,29]. In the present study, we observed that higher serum MDA-LDL levels were independently associated with PAS in HD patients, even with adjusting for potential confounders such as age, CRP, albumin, and pre-dialysis creatinine levels. This finding is consistent with previous studies reporting an association between MDA-LDL and arterial stiffness in other populations [13,21].

The mechanisms underlying the association between MDA-LDL and PAS in HD patients were likely multifactorial. MDA-LDL has been shown to promote endothelial dysfunction by increasing the expression of adhesion molecules, facilitating leukocyte recruitment, and impairing nitric oxide bioavailability [20,30,31]. Additionally, MDA-LDL may contribute to arterial stiffening by stimulating the proliferation and migration of vascular smooth muscle cells and promoting the synthesis of extracellular matrix components, such as collagen [13,32]. Moreover, the uremic milieu in ESRD patients may exacerbate the adverse effects of MDA-LDL on the arterial wall leading to accelerated arterial stiffening [33]. The ROC curve analysis identified a serum MDA-LDL level of 80.91 mg/dL as an optimal cut-off value for predicting the presence of PAS in our cohort of HD patients. Further prospective studies are needed to validate the predictive value of this cut-off level and to investigate the potential utility of serum MDA-LDL as a biomarker for risk stratification and management of PAS in HD patients.

As stated above, most of the prior studies on the association between various kinds of lipids such as modified LDLs and PAS and aortic wall stiffness were carried out in the general population [34,35,36,37,38,39], while there were rare studies conducted for HD patients [21]. Among HD patients, serum LDL levels are commonly normal or lower, whereas TG-rich lipoproteins levels are higher as compared to non-HD individuals [40]. Elevated TG levels, frequently found in CKD patients, could contribute to oxidative stress, oxLDL, vascular inflammation, and endothelial dysfunction which could be regarded as a critical precursor of PAS [41,42]. In a study conducted by An et al. [43], oxLDL to LDL ratio was identified as an independent predictor of vascular calcification in the feet, a representative of PAS, of HD patients [44]. Based on currently available evidence including our prior study for MDA-LDL and its association with increased aortic wall stiffness in HD patients, modified LDLs might be potential contributors to reducing both the large and small arterial elasticity in HD patients. While studies in non-HD populations have revealed that pharmacological interventions, such as statins, can reduce oxLDL, oxidative stress, and arterial stiffness [40], their effects on MDA-LDL levels and PAS in HD patients remain to be established.

Aging is a potent risk factor for PAS in the general population and in patients with chronic kidney disease (CKD) [45], and this association is also observed in HD patients. This association can be attributed to age-related changes in the arterial wall, including increased collagen content, decreased elastin content, and accumulation of glycation end-product [46]. The cumulative exposure to both traditional and non-traditional CVD risk factors in older HD patients may further contribute to the development of PAS [23]. Inflammation has been involved in the development of arterial stiffness and CVD events in CKD patients [42,47]. It has been well known that serum CRP may be a mediator of the development of CVD events and post-statin therapeutic CRP levels rather than LDL were associated with subsequent CVD events [48]. In our study, elevated serum CRP was independently associated with PAS in HD patients. This finding is consistent with many prior reports that chronic inflammation contributes to arterial stiffening in HD patients [46,49]. Modified LDLs have been found to cause vascular inflammation and thus increase serum CRP levels [50,51]. In a systemic review, a mild to moderate correlation between CRP levels and PWV (Pearson r = 0.33 to r = 0.624) was observed in individuals with dyslipidemia in most of the relevant studies [52]. As the levels of various modified LDLs may be increased in advanced CKD or HD patients, the association of CRP levels with PAS independent of MDA-LDL was possibly related to increases in other modified LDLs which were not assessed in this study. The mechanisms linking inflammation to PAS in HD patients are complex and involve the direct effects of pro-inflammatory cytokines on the arterial wall, as well as the indirect effects of inflammation on oxidative stress, endothelial dysfunction, and vascular calcification [53]. A combination of serum levels of MDA-LDL and CRP may be a good target to assess the status of PAS in HD patients in future studies.

The potential therapeutic implication of our findings merits more consideration. Strategies aimed at reducing oxidative stress and inflammation levels may be effective in preventing or slowing the progression of PAS among HD patients. Antioxidant therapies, such as vitamin E supplementation, have demonstrated promise in reducing CVD risk in some populations [54], although their efficacy in HD patients remains to be established. Similarly, anti-inflammatory agents, e.g., statins and angiotensin-converting enzyme inhibitors, have been shown to improve PAS in CKD patients [55], while their beneficial effects in HD patients require further investigation. Lifestyle modifications, e.g., regular exercise and dietary interventions, may reduce oxidative stress and chronic inflammation in HD patients. Exercise training has been shown to improve PAS and endothelial function in CKD patients [56], and dietary approaches, e.g., the Mediterranean diet, have been associated with reduced chronic inflammation and improved CVD outcomes in the general population [57]. However, the optimal exercise regimen and dietary strategy for preventing PAS in HD patients remain to be determined.

Several limitations of this study should be acknowledged. First, the cross-sectional design precludes the establishment of a causal relationship between serum MDA-LDL levels and PAS. Prospective studies are needed to confirm our findings and to evaluate the prognostic value of serum MDA-LDL levels in predicting CVD events in HD patients. Second, the relatively small sample size and single-center setting may limit the generalizability of our results. Larger, multicenter studies are warranted to validate our findings. Finally, we did not assess other biomarkers of oxidative stress or inflammation, which may have provided additional insights into the pathogenesis of PAS in this population.

## 5. Conclusions

Our study demonstrated that higher serum MDA-LDL, older age, and elevated serum CRP were independently associated with PAS in HD patients. Moreover, serum MDA-LDL levels ≥80.91 mg/dL may serve as a useful biomarker for predicting PAS in this population. The mechanisms linking inflammation to PAS in HD patients are complex and involve the direct effects of pro-inflammatory cytokines on the arterial wall, as well as the indirect effects of inflammation on oxidative stress, endothelial dysfunction, and vascular calcification. While our study demonstrates the independent associations of MDA-LDL and CRP with PAS in HD patients, further cohort studies with larger sample sizes and clinical trials are needed to evaluate the potential therapeutic implications of the findings to develop effective strategies for reducing CVD in this vulnerable population.

## Figures and Tables

**Table 1:** Clinical variables of chronic hemodialysis patients with baPWV ≤ 18.0 m/s or baPWV > 18.0 m/s.

Items	All Participants (*n* = 100)	baPWV ≤ 18 m/s Group (*n* = 48)	baPWV > 18 m/s Group (*n* = 52)	*p* Value
Age (years)	63.84 ± 13.51	57.94 ± 13.35	67.37 ± 12.11	<0.001 *
HD duration (months)	55.92 (21.96–123.60)	55.44 (19.44–132.00)	56.16 (24.42–112.62)	0.992
Height (cm)	160.70 ± 7.43	162.09 ± 9.19	159.47 ± 5.21	0.079
Pre-HD body weight (kg)	63.52 ± 15.13	66.37 ± 16.16	60.89 ± 13.74	0.070
Post-HD body weight (kg)	61.36 ± 14.68	64.05 ± 15.76	58.88 ± 13.29	0.079
Body mass index (kg/m^2^)	24.49 ± 5.20	25.16 ± 5.73	23.90 ± 4.66	0.226
Waist circumference (cm)	90.68 ± 12.48	90.40 ± 13.32	90.94 ± 11.76	0.828
Systolic blood pressure (mmHg)	140.49 ± 25.73	138.31 ± 24.81	142.50 ± 26.64	0.419
Diastolic blood pressure (mmHg)	76.60 ± 15.16	78.50 ± 15.33	74.85 ± 14.93	0.230
Left baPWV (m/s)	18.04 ± 3.25	15.47 ± 1.91	20.41 ± 2.28	<0.001 *
Right baPWV (m/s)	18.14 ± 3.29	15.38 ± 1.55	20.68 ± 2.26	<0.001 *
Hemoglobin (g/dL)	10.41 ± 1.18	10.36 ± 1.26	10.46 ± 1.11	0.652
Albumin (g/dL)	4.15 ± 0.43	4.25 ± 0.41	4.06 ± 0.43	0.022 *
Total cholesterol (mg/dL)	149.14 ± 35.87	145.88 ± 33.97	152.15 ± 37.61	0.384
Triglyceride (mg/dL)	153.91 ± 80.23	152.54 ± 75.19	155.17 ± 85.33	0.871
MDA-LDL (mg/dL)	88.67 (69.96–148.52)	78.38 (59.41–98.35)	119.67 (81.65–176.54)	<0.001 *
Glucose (mg/dL)	132.50 (110.00–171.25)	131.00 (104.00–172.00)	133.00 (113.00–173.00)	0.377
Blood urea nitrogen (mg/dL)	59.18 ± 14.21	61.71 ± 14.68	56.85 ± 13.49	0.087
Creatinine (mg/dL)	9.41 ± 1.87	9.85 ± 1.98	8.99 ± 1.67	0.021 *
Total calcium (mg/dL)	8.90 ± 0.75	8.86 ± 0.67	8.95 ± 0.83	0.549
Phosphorus (mg/dL)	4.55 ± 1.25	4.68 ± 1.28	4.43 ± 1.22	0.326
Intact parathyroid hormone (pg/mL)	204.05 (56.83–355.30)	205.20 (69.40–461.30)	192.90 (54.90–340.65)	0.297
C-reactive protein (mg/dL)	0.28 (0.06–0.66)	0.16 (0.05–0.40)	0.35 (0.10–1.01)	0.007 *
Urea reduction rate	0.74 ± 0.05	0.73 ± 0.05	0.74 ± 0.04	0.181
Kt/V (Gotch)	1.36 ± 0.19	1.33 ± 0.21	1.38 ± 0.18	0.195
Female, *n* (%)	47 (47.0)	18 (38.3)	29 (54.7)	0.101
Diabetes mellitus, *n* (%)	46 (46.0)	19 (40.4)	27 (50.9)	0.292
Hypertension, *n* (%)	46 (46.0)	22 (46.8)	24 (45.3)	0.879
Angiotensin receptor blocker, *n* (%)	28 (28.0)	15 (31.9)	13 (24.5)	0.412
β-blocker, *n* (%)	35 (35.0)	15 (31.9)	20 (37.7)	0.542
Calcium channel blocker, *n* (%)	40 (40.0)	21 (44.7)	19 (35.8)	0.368
Statin, *n* (%)	31 (31.0)	13 (27.7)	18 (34.0)	0.496
Fibrate, *n* (%)	25 (25.0)	12 (25.5)	13 (24.5)	0.908

Values for continuous variables are shown as mean ± standard deviation after analysis by Student’s *t*-test; variables not normally distributed are shown as median and interquartile range after analysis by the Mann-Whitney U test; values are presented as number (%) and analysis after analysis by the chi-square test. HD, hemodialysis; MDA-LDL, malondialdehyde-modified low-density lipoprotein, Kt/V, fractional clearance index for urea. * *p* < 0.05 was considered statistically significant.

**Table 2:** Multivariable logistic regression analysis of the factors correlated to peripheral arterial stiffness among chronic hemodialysis patients.

Variables	Odds Ratio	95% Confidence Interval	*p* Value
MDA-LDL, 1 mg/mL	1.014	1.004–1.025	0.009 *
Age, 1 year	1.044	1.004–1.085	0.031 *
C-reactive protein, 1 mg/dL	3.697	1.149–11.893	0.028 *
Albumin, 1 g/dL	0.526	0.144–1.926	0.332
Creatinine, 1 mg/dL	0.971	0.734–1.285	0.837

MDA-LDL, malondialdehyde-modified low-density lipoprotein. * *p* < 0.05 was considered statistically significant in the multivariate logistic regression analysis (adopted factors: age, albumin, creatinine, C-reactive protein, and MDA-LDL).

**Table 3:** Spearman correlation coefficients between left baPWV, right baPWV, log-transformed malondialdehyde-modified low-density lipoprotein, and clinical variables in chronic hemodialysis patients.

Variables	Left baPWV (m/s)		Right baPWV (m/s)	
	Spearman’s Coefficient of Correlation	*p* Value	Spearman’s Coefficient of Correlation	*p* Value
Age (years)	0.284	0.004 *	0.308	0.002 *
Log-HD duration (months)	–0.037	0.711	0.009	0.930
Height (cm)	–0.115	0.256	–0.136	0.178
Pre-HD body weight (kg)	–0.168	0.094	–0.147	0.146
Body mass index (kg/m^2^)	–0.146	0.146	–0.115	0.253
Waist circumference (cm)	0.003	0.973	0.029	0.778
Systolic blood pressure (mmHg)	0.121	0.232	0.119	0.238
Diastolic blood pressure (mmHg)	–0.070	0.487	–0.096	0.343
Left baPWV (m/s)	—	—	0.875	<0.001 *
Right baPWV (m/s)	0.875	<0.001 *	—	—
Hemoglobin (g/dL)	0.053	0.601	0.054	0.593
Albumin (g/dL)	–0.160	0.111	–0.180	0.072
Total cholesterol (mg/dL)	0.025	0.806	0.060	0.553
Triglyceride (mg/dL)	0.075	0.461	0.021	0.835
Log-MDA-LDL (mg/dL)	0.385	<0.001 *	0.390	<0.001 *
Log-Glucose (mg/dL)	0.227	0.023 *	0.191	0.054
Blood urea nitrogen (mg/dL)	–0.126	0.212	–0.101	0.317
Creatinine (mg/dL)	–0.221	0.027 *	–0.216	0.031 *
Total calcium (mg/dL)	–0.100	0.323	–0.031	0.758
Phosphorus (mg/dL)	–0.099	0.329	–0.070	0.466
Log-iPTH (pg/mL)	–0.184	0.067	–0.085	0.403
Log-CRP (mg/dL)	0.307	0.002 *	0.249	0.013 *
Urea reduction rate	0.184	0.067	0.106	0.292
Kt/V (Gotch)	0.195	0.052	0.154	0.127

Data of glucose, C-reactive protein, iPTH, and MDA-LDL levels showed skewed distribution and, therefore, were log-transformed before analysis. HD, hemodialysis; baPWV, brachial-ankle pulse wave velocity; MDA-LDL, malondialdehyde-modified low-density lipoprotein, iPTH, intact parathyroid hormone; CRP, C-reactive protein, Kt/V, fractional clearance index for urea. * *p* < 0.05 was considered statistically significant (2-tailed).

**Table 4:** The optimal cut-off value of serum MDA-LDL and related diagnostic performances for predicting peripheral arterial stiffness among chronic hemodialysis patients.

Cutoff	Sensitivity	Specificity	PPV	NPV
80.91 mg/dL	79.25%	59.57%	68.85%	71.80%

MDA-LDL, malondialdehyde-modified low-density lipoprotein; NPV, negative predictive value; PPV, positive predictive value.

## Data Availability

Upon request, the corresponding author can provide the data utilized in this study.
